# Polydopamine-coated UiO-66 nanoparticles loaded with perfluorotributylamine/tirapazamine for hypoxia-activated osteosarcoma therapy

**DOI:** 10.1186/s12951-021-01013-0

**Published:** 2021-09-30

**Authors:** Hongfang Chen, You Fu, Kai Feng, Yifan Zhou, Xin Wang, Haohan Huang, Yan Chen, Wenhao Wang, Yuanjing Xu, Haijun Tian, Yuanqing Mao, Jinwu Wang, Zhiyuan Zhang

**Affiliations:** 1 Shanghai Key Laboratory of Orthopaedic Implants, Department of Orthopaedic Surgery, Shanghai Ninth People’s Hospital, Shanghai Jiao Tong University School of Medicine, Shanghai, 200011 China; 2 Department of Oral & Maxillofacial - Head & Neck Oncology, Shanghai Ninth People’s Hospital, Shanghai Jiao Tong University School of Medicine; College of Stomatology, Shanghai Jiao Tong University; National Center for Stomatology; National Clinical Research Center for Oral Diseases; Shanghai Key Laboratory of Stomatology , Shanghai, China; 3 Institute of Microsurgery on Extremities, Department of Orthopedic Surgery, Shanghai Jiaotong University Affiliated Sixth People’s Hospital, Shanghai, China; 4 College of Medicine, Southwest Jiaotong University, Chengdu, China; 5 School of Biomedical Engineering, Shanghai Jiao Tong University, Shanghai, China

**Keywords:** Metal-organic framework (MOF), Tumor hypoxia, Photothermal therapy (PTT), Osteosarcoma

## Abstract

**Background:**

Hypoxia is a characteristic of solid tumors that can lead to tumor angiogenesis and early metastasis, and addressing hypoxia presents tremendous challenges. In this work, a nanomedicine based on oxygen-absorbing perfluorotributylamine (PFA) and the bioreductive prodrug tirapazamine (TPZ) was prepared by using a polydopamine (PDA)-coated UiO-66 metal organic framework (MOF) as the drug carrier.

**Results:**

The results showed that TPZ/PFA@UiO-66@PDA nanoparticles significantly enhanced hypoxia, induced cell apoptosis in vitro through the oxygen-dependent HIF-1α pathway and decreased oxygen levels in vivo after intratumoral injection. In addition, our study demonstrated that TPZ/PFA@UiO-66@PDA nanoparticles can accumulate in the tumor region after tail vein injection and effectively inhibit tumor growth when combined with photothermal therapy (PTT). TPZ/PFA@UiO-66@PDA nanoparticles increased HIF-1α expression while did not promote the expression of CD31 in vivo during the experiment.

**Conclusions:**

By using TPZ and PFA and the enhanced permeability and retention effect of nanoparticles, TPZ/PFA@UiO-66@PDA can target tumor tissues, enhance hypoxia in the tumor microenvironment, and activate TPZ. Combined with PTT, the growth of osteosarcoma xenografts can be effectively inhibited.

**Graphic abstract:**

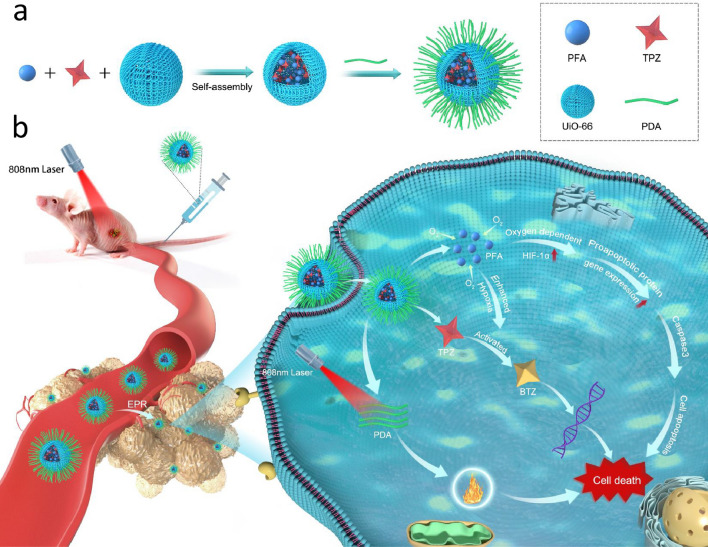

**Supplementary Information:**

The online version contains supplementary material available at 10.1186/s12951-021-01013-0.

## Introduction

The fast growth and insufficient nutrient supply of solid tumors result in tumor hypoxia [1, 2], which can not only promote tumor angiogenesis and metastasis [3] but can also lead to therapy resistance [4, 5]. To treat hypoxic tumors, two methods are highlighted: tumor oxygenation and tumor deoxygenation [6–8]. For tumor oxygenation, various oxygen-delivery strategies have been explored to alleviate tumor hypoxia [9, 10]. However, the therapeutic effect of these solutions can be unsatisfactory due to premature O_2_ leakage, low O_2_ production, and insufficient neovascularization and blood flow in tumor tissues. Hence, strategies focused on tumor deoxygenation combined with hypoxia-activated prodrugs have also been developed for hypoxic tumor treatment.

Tirapazamine (TPZ) is a bioreductive prodrug that can be transformed into tumor-toxic benzotriazinyl (BTZ) by hypoxia [11]. However, a single use of TPZ can elicit only a marginal effect on tumor growth because a limited amount of BTZ is generated from TPZ due to restricted penetration into the tumor and insufficient tumor hypoxia [12]. Accordingly, many studies have focused on creating a penetrable and hypoxic intratumor microenvironment to enhance the toxicity of hypoxia-sensitive prodrugs [13–15]. Perfluorotributylamine (PFA) is a clinically approved perfluorocarbon that can enhance tumor permeability and deoxygenation due to its platelet inhibition and oxygen-absorbing effects. Wang et al. [16] demonstrated that PFA can establish a long-lasting favorable tumor microenvironment to enable hypoxia-sensitive prodrug delivery and activation.

Metal organic frameworks (MOFs) are a new class of topologically organic-inorganic hybrid materials constructed by inorganic nodes and organic ligands via spontaneous self-assembly [17] and bear multiple complexing functions [18]. The large family of organic linkers available [19] present high and regular porosities, allowing MOFs to be used for gas adsorption, separation and storage, molecular sensing, drug delivery, pollution management, catalysis, and energy storage [20–23]. MOFs can simultaneously exhibit hydrophobic and hydrophilic properties and have a tunable connectivity and pore size; thus, MOFs can be specifically adapted to the physico-chemical properties of each drug and its medical application [24, 25]. For example, glucose/oxygen depletion agents, such as glucose oxidase and gold nanoparticles (NPs), can be integrated into MOF NPs to promote intracellular Fenton-like reactions and effectively suppress tumor growth [26, 27]. Since the pore size, pore shape and pore chemistry of MOF materials can be precisely controlled, numerous MOFs have been developed for gas adsorption. Chen et al. [28] synthesized a novel mesoporous MOF constructed from a trigonal tetrazolate ligand and Cu^2+^, which demonstrated exceptionally high uptake capacities for fluorocarbons due to its high porosity. Shen et al. [29] demonstrated that sulfone-based 2D fluorinated MOFs showed a high adsorption capacity with high selectivity and stability, enabling the separation of acetylene from ethylene. The characteristics of MOF materials make them appealing drug carriers for cancer treatment. In this study, we used PFA-loaded MOF NPs as an oxygen absorbent to promote tumor hypoxia. Since hypoxia is often associated with therapeutic resistance, the combination of other therapeutic methods was considered. Photothermal therapy (PTT) has been developed in the past decade and is regarded as a promising strategy due to its noninvasiveness, limited side effects, and high ablation efficacy [30]. PTT is utilized to further enhance tumor permeability and the killing of drug-resistant tumor cells.

**Scheme 1:**
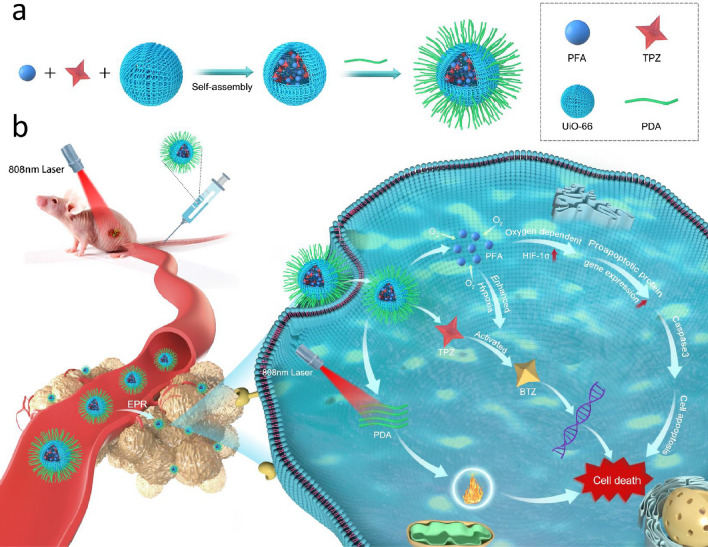
Schematic illustration of **a** the preparation of TPZ/PFA@UiO-66@PDA NPs and **b** tail vein-injected TPZ/PFA@UiO-66@PDA NPs combined with laser radiation for hypoxia-activated osteosarcoma therapy

In this study, we developed a microporous MOF-based nanocarrier coated with polydopamine (PDA) and encapsulating PFA and TPZ for hypoxia-activated osteosarcoma therapy. As illustrated in Scheme 1, UiO-66 was chosen as a nanocarrier to encapsulate PFA and the prodrug TPZ because of its large surface area and high porosity. Then, the fabricated MOF NPs were further coated with PDA to obtain TPZ/PFA@UiO-66@PDA. After intravenous injection, TPZ/PFA@UiO-66@PDA effectively accumulated in tumor tissue via the typical enhanced permeability and retention (EPR) effect. The PFA in TPZ/PFA@UiO-66@PDA efficiently absorbed oxygen to aggravate the hypoxic tumor microenvironment. Enhanced tumor hypoxia could then activate and transform the prodrug TPZ into highly cytotoxic radicals to induce tumor cell apoptosis. Moreover, the accumulation and internalization of PDA further enhanced tumor permeability and the killing of drug-resistant tumor cells under 808 nm laser irradiation. In this way, TPZ/PFA@UiO-66@PDA NPs achieved synergistic efficacy between hypoxia-activated bioreductive prodrug therapy and PTT to effectively suppress tumor growth.

## Experiments

### Ethics statement

All experiments involving animals were reviewed and approved by the Ethical Committee of Shanghai Ninth People’s Hospital in Shanghai, China.

### Synthesis of drug-loaded UiO-66

UiO-66 was synthesized based on a previously reported method [31–33]. ZrCl_4_ (1 mmol or 0.2332 g) and TPA (1 mmol or 0.161 g) were dissolved in 50 mL of DMF solution and then transferred to a 100 mL autoclave. The autoclave was sealed and heated in an oven at 120 °C for 48 h. The resulting white powder was washed several times with methanol after cooling and then dried at 100 °C for 12 h under vacuum. Finally, the powder was stored for further use. To load the anticancer drugs into the MOF, 100 mg of UiO-66 and 50 mg of TPZ were poured into a mixture of 5 mL of DMSO and 5 ml of ethanol, stirred for 12 h, and then centrifuged at 5000 rpm for 20 min. The mixture was washed with ethanol twice and freeze dried. Then, 100 mg of the mixture was poured into 0.2 mL of PFA, placed on a shaker for 3 h, and centrifuged. Next, 100 mg of the mixture and 35 mg of PDA were poured into 20 mL of hydrochloric acid with a pH of 8.5, stirred for 45 min, and then centrifuged at 5000 rpm for 20 min. After freeze-drying, TPZ/PFA@UiO-66@PDA NPs were dispersed in phosphate-buffered saline (PBS) for further use. For IR780-doped PFA/TPZ@UiO-66@PDA NPs, IR780 (5 mg) and PFA/TPZ@UiO-66@PDA (50 mg) were mixed in ethanol for 24 h. After centrifugation, the mixture was dispersed in 20 mL Tris-HCl (pH = 8.5) and stirred for 45 min. Then, the mixture was centrifuged and freeze dried for further use.

### General characterization

The crystallinity and surface morphology of the prepared UiO-66 MOF were detected using X-ray diffraction (XRD, D8 VENTURE), Brunauer-Emmett-Teller (BET) surface area analysis (Autosorb-IQ3), scanning electron microscopy (SEM, MIRA3), and transmission electron microscopy (TEM, Talos F200X G2). UiO-66 and UiO-66@PDA were characterized by Fourier Transform Infrared Spectroscopy (FTIR). The drug loading efficiency of UiO-66, TPZ@UiO-66, and TPZ/PFA@UiO-66 was analyzed via thermogravimetric analysis (TGA, Pyris 1). The particle size distribution of UiO-66, UiO-66@PDA and TPZ/PFA@UiO-66@PDA were detected by dynamic light scattering (DLS).

### Cell culture

143B cells were obtained from ATCC (Manassas, VA) and used in this study. The cells were cultured in Dulbecco’s minimum essential medium (DMEM) supplemented with 10% fetal bovine serum (FBS), penicillin (100 U/mL), and streptomycin (100 mg/mL) in a humidified atmosphere containing 5% CO_2_ at 37 °C. The culture medium was changed every 2 days. Cells were passaged upon reaching 80–90% confluence.

### Cell viability

Cell viability was assessed using a live/dead assay (Thermo Fisher Scientific). 143B cells were divided into the following groups: control, TPZ (40 µg mL^− 1^), TPZ@UiO-66 (40 µg mL^− 1^), TPZ/PFA@UiO-66@PDA (40 µg mL^− 1^), and TPZ/PFA@UiO-66@PDA + 808 nm laser radiation. Each group was cultured for 3 h in 24-well plates at a density of 2.5 × 10^4^ cells/well. For group 5, cells were irradiated with an 808 nm laser for 5 min. Then, all samples were stained in a solution containing calcein-AM (2 µM in PBS) and ethidium homodimer (EthD)-1 (4 µM) for 30 min and examined using a fluorescence microscope (Leica, Wetzlar, Germany). Live cells were stained green, and dead cells were stained red.

### In vitro hypoxia and apoptosis analysis using flow cytometry (FCM)

143B cells were seeded into 6-well plates and cultured. When a confluence of 70–80% was reached, the cells were washed with PBS and grouped as previously stated. For apoptosis analysis, 5 µL of annexin V-FITC and 5 µL of propidium iodide (PI) were added to all wells and then incubated with the cells for 15 min at room temperature in the dark according to the manufacturer’s instructions (Thermo Scientific, Waltham, MA, USA). For hypoxia analysis, cell suspensions of each group were treated with 100 µM Hypoxyprobe-1 (pimonidazole hydrochloride) for 1 h. The cells were collected, fixed and stained with anti-pimonidazole antibody (1:200, clone 4.3.11.3; FITC-MAb1). The fluorescence intensity of all samples was immediately analyzed via flow cytometry.

### Quantitative real-time PCR

To examine the effects of the NPs on oxygen-dependent signaling in 143B cells at the gene level, 143B cells were seeded and grouped as follows: Control, TPZ (40 µg mL^− 1^), TPZ@UiO-66 (40 µg mL^− 1^), and TPZ/PFA@UiO-66@PDA (40 µg mL^− 1^). All groups were cultured in 6-well plates at a density of 1 × 10^5^ cells/well for 3 h. Total RNA was isolated using an E.Z.N.A.® HP Total RNA Kit (Omega Bio-Tek, Norcross, GA, USA). One microgram of RNA was reverse transcribed using a Prime-Script RT reagent kit (Takara, Shiga, Japan). Real-time PCR was performed in a 7300 Real-Time PCR system using SYBR® Premix Ex Taq™ (Takara, Shiga, Japan). The primer sequences used in this study are listed in Table 1. Glyceraldehyde 3-phosphate dehydrogenase 1 (GAPDH) was evaluated for each RT reaction as a standard. The ΔΔCT method was used to analyze relative gene expression.

**Table 1:** Sense and anti-sense primers for real-time PCR

Gene	Forward (5′ to 3′)	Reverse (5′ to 3′)
HIF-1α	AGTTCCGCAAGCCCTGAAAGC	GCAGTGGTAGTGGTGGCATTAGC
HIF-1β	GCTACTGCTAAGACTCGTACTT	ACGATTGGTGAGACTAGGGTAG
p300	GTTCCTTCCTCAGACTCAGTTC	CATTATAGGAGAGTTCACCGGG
CBP	CAAGGAGGTCTTCTTCGTGATC	CTCGTTGCAGGTGTAGACAAAG
BAX	CGAACTGGACAGTAACATGGAG	CAGTTTGCTGGCAAAGTAGAAA
BAK	AGAGATGGTCACCTTACCTCT	GGTCTGGAACTCTGAGTCATAG
BID	CTGTGAACCAGGAGTGAGTC	GTAGGTTTGTGATGCACTCATC
Caspase-3	CCAAAGATCATACATGGAAGCG	CTGAATGTTTCCCTGAGGTTTG
GAPDH	CAGCGACACCCACTCCTC	TGAGGTCCACCACCCTGT

### Western blotting analysis

To examine the effects of NPs on HIF-1α in 143B cells at the protein level, 143B cells were seeded and grouped as follows: Control, TPZ (40 µg mL^− 1^), TPZ@UiO-66 (40 µg mL^− 1^), and TPZ/PFA@UiO-66@PDA (40 µg mL^− 1^). All groups were cultured for 3 h in 6-well plates at a density of 1 × 10^5^ cells/well. The cells were washed three times with PBS and then lysed with RIPA buffer (150 mM NaCl, 1% sodium deoxycholate, 0.1% SDS, 50 mM Tris-HCl pH 7.4, 1 mM EDTA, 1 mM PMSF, and 1% Triton X-100) supplemented with protease inhibitors and phosphatase inhibitors for 30 min at 4 °C. A total of 20–30 mg of protein was separated via 10% sodium dodecyl sulfate-polyacrylamide gel electrophoresis and electrotransferred onto nitrocellulose membranes. A rabbit polyclonal anti-HIF-1α antibody (Cell Signaling Technology) was used as the primary antibody. GAPDH antibody (Cell Signaling Technology) was used to normalize protein loading. The protein bands were visualized using an Odyssey Infrared Imaging System (LI-COR Biosciences, Lincoln, NE, USA).

### Implantation of tumor cells and test materials

Six-week-old male BALB/c nude mice were purchased from Shanghai Super-B&K Laboratory Animal Corp. Ltd. A total of 1.0 × 10^6^ 143B cells in a volume of 100 µL that contained 50 µL of culture medium and 50 µL of growth factor-reduced Matrigel (BD Biosciences, San Jose, CA, USA) were subcutaneously injected into the lower left flanks of the mice. When the tumor size reached approximately 80 mm^3^, the mice were divided into five groups as follows: Group 1, Control group treated with saline only; Group 2, TPZ (10 mg kg^− 1^, 100 µL); Group 3, TPZ@UiO-66 (10 mg kg^− 1^, 100 µL); Group 4, TPZ/PFA@UiO-66@PDA (10 mg kg^− 1^, 100 µL); and Group 5, TPZ/PFA@UiO-66@PDA + 808 nm laser radiation. The treatment or saline was intravenously injected into the mice on days 0, 4 and 8. The tumors in group 5 were irradiated three times (808 nm, 1.5 W cm^− 2^) for 5 min at 24 h after intravenous injection. The mice were anesthetized and intraperitoneally injected with D-luciferin (150 mg/kg, Caliper Life Sciences, Hopkinton, MA). Twenty minutes after the injection, all mice were photographed using an IVIS cooled CCD camera (Xenogen, Alameda, CA). The mice were imaged on days 0, 8 and 16. The weights of all mice were measured every other day. The dissected tumor weight was measured using a precision electronic balance.

### In vivo photoacoustic (PA) imaging

For in vivo PA imaging, 143B tumor-bearing mice were intratumorally injected with NPs (10 mg kg^− 1^, 100 µL). The intensities of PA signals and PA images of tumor-bearing mice were measured and recorded at different time points (0, 0.5, 1, 2, and 3 h). The intensities of the PA signals and PA images from mice injected with saline were used as the control. The imaging parameters were set as follows: PA gain, 47 dB, and 2D gain, 20 dB.

### Histology and immunohistochemistry

143B cells were cultured in 10 mm confocal dishes at a density of 1 × 10^5^ cells/dish. After cell attachment, the dishes were treated and grouped as follows: Control, TPZ, TPZ@UiO-66, and TPZ/PFA@UiO-66@PDA. Each group was incubated for 3 h. Then, all dishes were rinsed with PBS, fixed for 15 min at room temperature in 4% paraformaldehyde (Sigma-Aldrich, St. Louis, MO, USA), and permeabilized with 0.2% Triton X-100. After blocking with 0.5% BSA and 1% FBS, the cells were stained with anti-HIF-1α antibody, followed by Alexa 488-conjugated secondary antibody. Finally, the samples were washed with PBS for direct observation under confocal laser scanning microscopy (Nikon Eclipse CI, Tokyo, Japan). The subcutaneous tumor specimens were fixed at 4 °C in 4% paraformaldehyde (Sigma-Aldrich, St. Louis, MO, USA) and washed in running water overnight before being embedded in paraffin. Hematoxylin-eosin (H&E) staining was conducted on 5-µm sections for histological analyses. Immunohistochemistry was performed to detect PCNA, CD31, HIF-1α and pimonidazole in tumor sections. Supplementary methods were provided in Additional file 1.

### Statistical analysis

In this article, all data are presented as the mean ± standard deviation (SD) and were analyzed with SPSS version 22.0 (IBM, Armonk, NY). Comparisons of two or more groups were performed using one-way analysis of variance (ANOVA) with subsequent Tukey-Kramer comparisons. Student’s t-test was used for comparisons involving two groups. P < 0.05 was considered statistically significant.

## Results and discussion

### Characterization of prepared UiO-66 and UiO-66/PDA

**Fig. 1:**
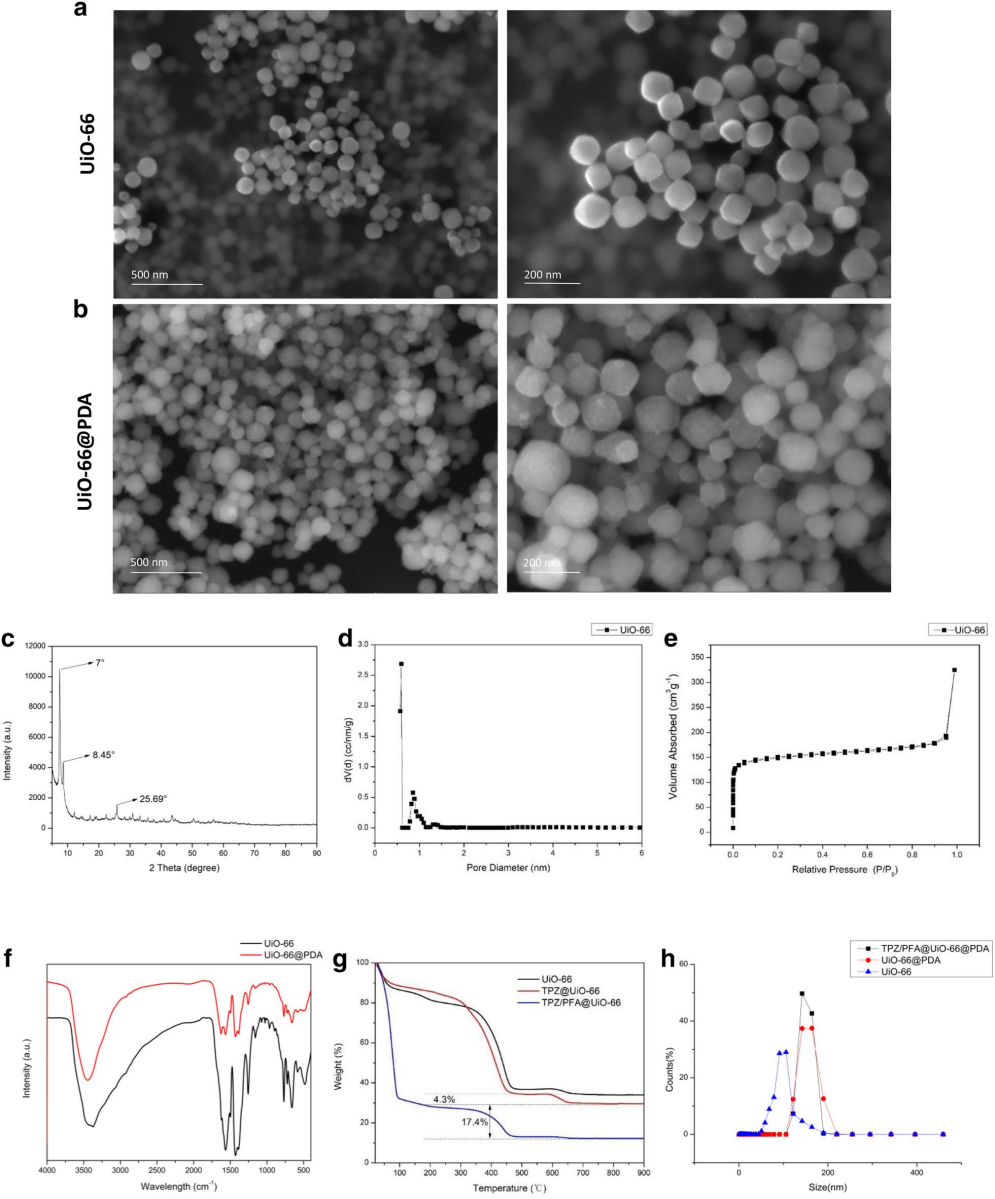
**a** SEM images of UiO-66. **b** SEM images of UiO-66@PDA. **c** XRD spectra of UiO-66. **d** BET surface area of UiO-66. **e** Pore volume of UiO-66. **f** FTIR spectra of UiO-66 and UiO-66@PDA. **g** TGA of UiO-66, TPZ@UiO-66, and TPZ/PFA@UiO-66. **h** DLS results of UiO-66, UiO-66@PDA and TPZ/PFA@UiO-66@PDA

**Fig. 2:**
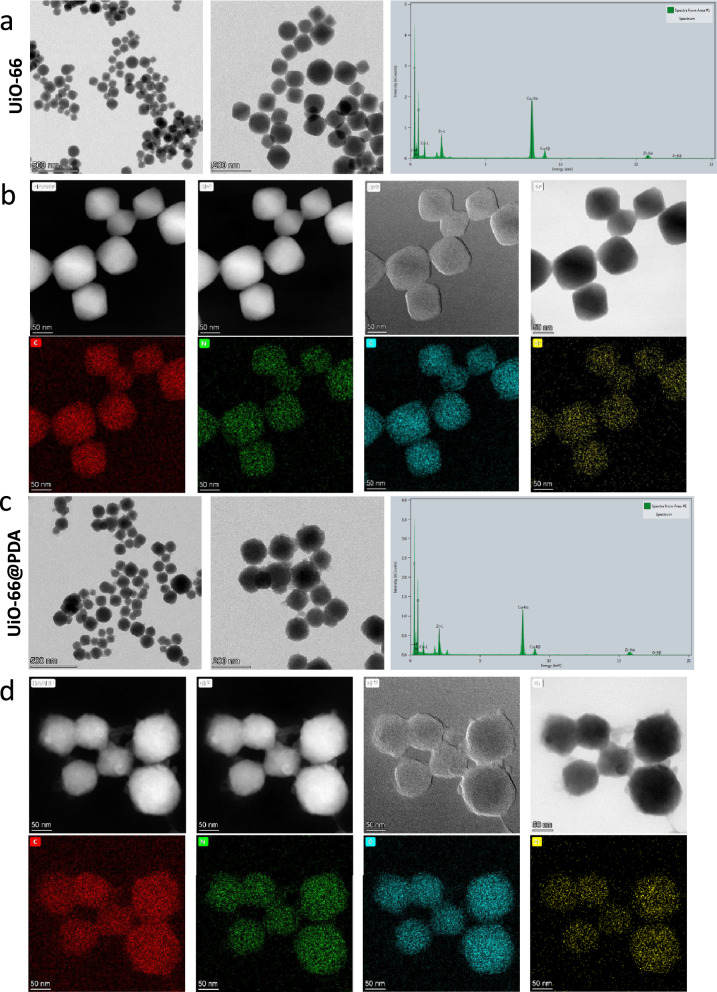
**a** TEM and EDS images of UiO-66. **b** Elemental mapping images of UiO-66. **c** TEM and EDS images of UiO-66@PDA. **d** Elemental mapping images of UiO-66@PDA

XRD and PXRD were employed to characterize the structure of the synthesized UiO-66. As shown in Fig. 1c, the XRD pattern of UiO-66 has three peaks at 7°, 8.45°, and 25.69° [33, 34]. Additional file 2: Fig. S1 showed the PXRD pattern of UiO-66. The Bragg diffraction peaks of the samples indicated that the NPs maintained a well-ordered pore structure, which was beneficial for drug delivery. BET analysis demonstrated that the UiO-66 samples displayed typical Type-I gas sorption isotherms. UiO-66 exhibited a BET surface area of 655.89 m^2^ g^− 1^, which indicated that the NPs were suitable for drug loading (Fig. 1d). Figure 1e showed that the pore volume was 0.274 cc/g. Figure 1f demonstrates the FTIR spectra of UiO-66 and UiO-66@PDA. The shapes of UiO-66 and UiO-66@PDA were characterized by SEM and TEM (Figs. 1a, b and 2a and c). Figures 1a and 2a showed the morphology of the prepared UiO-66. The TEM image demonstrated that the sizes of the most UiO-66 crystals were between 80 and 160 nm. Elemental mapping images revealed that Zr was dispersed homogeneously in UiO-66 (Fig. 2b). The SEM and TEM images of TPZ/PFA@UiO-66@PDA were provided in Additional file 3: Fig. S2. After modification with PDA, the UiO-66@PDA composite displayed rough surfaces (Fig. 2c). Figure 2d showed the elemental mapping of UiO-66@PDA. These results indicated successful fabrication of UiO-66@PDA NPs and that the coating process did not change the elemental distribution of UiO-66. TPZ/PFA@UiO-66@PDA NPs remained stable in PBS or culture media for at least 24 h (Additional file 4: Fig. S3). Figure 1g showed that the mass fraction of TPZ in TPZ@UiO-66 was 4.3%, and the mass fraction of PFA in TPZ/PFA@UiO-66 was 17.4%, and the drug-loading efficiency for TPZ and PFA of UiO-66 was 8.6 and 7.062% respectively. Additional file 5: Fig. S4 demonstrated the standard calibration curve of TPZ and the in vitro release curve of TPZ from TPZ@UiO-66 and TPZ/PFA@UiO-66@PDA. And TPZ/PFA@UiO-66@PDA NPs showed a slow release pattern of TPZ. DLS results of UiO-66, UiO-66@PDA and TPZ/PFA@UiO-66@PDA indicated that the particle size increased after PDA coating and drug loading (Fig. 1h). TPZ/PFA@UiO-66@PDA NPs remained stable in PBS or culture media for at least 24 h (Fig. S3).

### Evaluation of TPZ/PFA@UiO-66@PDA in cell culture

**Fig. 3:**
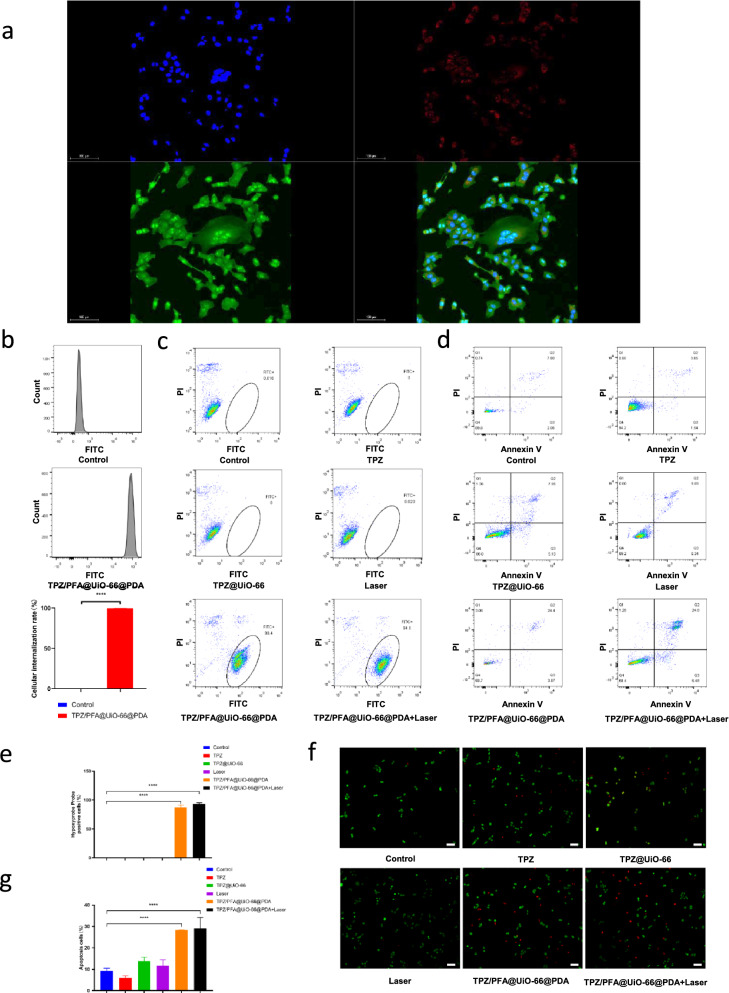
**a** CLSM images of 143B cells incubated with PFA/TPZ@UiO-66@PDA NPs for 3 h. Red fluorescence is attributed to IR780-doped PFA/TPZ@UiO-66@PDA NPs. Nuclei were stained blue with DAPI. Cytoskeletons were stained green with FITC-Phalloidin. **b** Cellular uptake behaviors of PFA/TPZ@UiO-66@PDA NPs determined by flow cytometry. **c** Hypoxia assessment via flow cytometry analysis of 143B cells under different treatments. **d** Apoptosis analysis of 143B cells after different treatments for 3 h. **e** The quantitative results for 143B cells obtained using pimonidazole hydrochloride.  f Live/dead staining of 143B cells after incubation with different treatments for 3 h. Viable cells were stained with calcein-AM (green), and dead/late apoptotic cells were stained with ethidium homodimer (EthD)-1 (red). **g **The quantitative flow cytometry results for cell apoptosis analysis. Asterisks indicate significant differences (scale bar, 100 μm) (*P < 0.05, **P < 0.01, ***P < 0.001, **** P < 0.0001)

Additional file 6: Fig. S5 indicated that UiO-66 showed no obvious toxicity in both 143B and rat bone marrow stem cells with different concentration of UiO-66 (0 *µ*g mL^−1^ , 10 *µ*g mL^−1^ , 20 *µ*g mL^−1^ , 40 *µ*g mL^−1^ , 80 *µ*g mL^−1^).To evaluate the cellular uptake behavior, TPZ/PFA@UiO-66@PDA NPs were incubated with 143B cells for 3 h. As illustrated in Fig. 3a, the red fluorescence in the cells suggested strong cellular internalization. Flow cytometry analysis also confirmed the high uptake of TPZ/PFA@UiO-66@PDA NPs (Fig. 3b). Then, we examined the O_2_-absorbing capacity of TPZ/PFA@UiO-66@PDA NPs in vitro. 143B cells were cultured with different treatments for 3 h. A hypoxia probe was used to detect enhanced cellular hypoxia. Flow cytometry and quantitative analysis showed significantly more pimonidazole hydrochloride-positive cells in the TPZ/PFA@UiO-66@PDA and TPZ/PFA@UiO-66@PDA + laser groups, confirming that TPZ/PFA@UiO-66@PDA NPs induced cellular hypoxia (Fig. 3c and e). To explore whether the TPZ/PFA@UiO-66@PDA NPs could improve the efficacy of tumor treatment, Annexin V/propidium iodide (PI) apoptosis detection and live/dead assays were performed to measure the apoptosis-inducing capability in vitro. Flow cytometry analysis showed that more apoptotic cells were found in the TPZ/PFA@UiO-66@PDA and TPZ/PFA@UiO-66@PDA + laser groups (Fig. 3d, g). Cells treated with TPZ/PFA@UiO-66@PDA and TPZ/PFA@UiO-66@PDA + laser displayed strong red fluorescence, whereas the remaining groups showed relatively weak red fluorescence (Fig. 3f). Additional file 7: Fig. S6 also demonstrated tumor cell growth was inhibited in the TPZ/PFA@UiO-66@PDA and TPZ/PFA@UiO-66@PDA+laser groups using Cell Counting Kit-8 method.

### TPZ/PFA@UiO-66@PDA upregulated the oxygen-dependent HIF-1α pathway and decreased O_2_ in tumor tissues

**Fig. 4:**
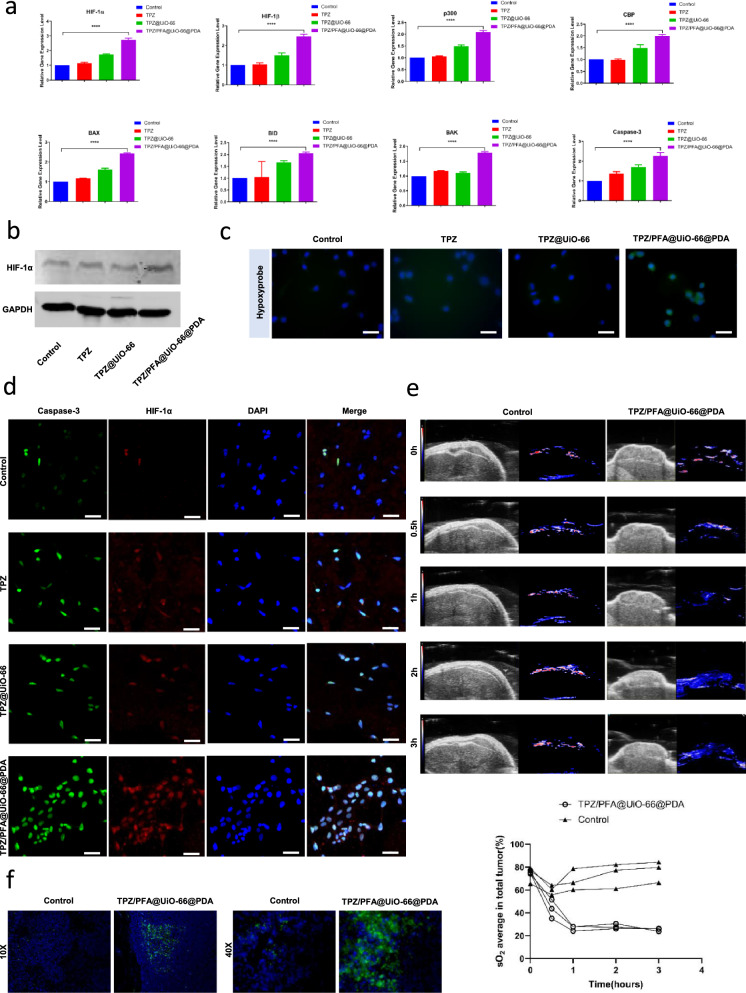
**a** The relative HIF-1α, HIF-1β, p300, CBP, BAX, BAK, BID and Caspase-3 gene expression levels in 143B cells subjected to different treatments for 3 h. **b** The HIF-1α protein expression level in 143B cells subjected to different treatments for 3 h. **c** Immunohistochemistry to detect hypoxia in 143B cells using the hypoxyprobe. **d** The expression of HIF-1α and Caspase-3 in 143B cells subjected to different treatments for 3 h analyzed by the double immunofluorescence labeling method. **e** In vivo photoacoustic images and the average oxygen content of tumor tissues at different time points after intratumoral administration of PFA/TPZ@UiO-66@PDA NPs or saline and the corresponding quantitative analysis. **f** Immunohistochemistry to detect HIF-1α in tumor sections at 24 h after intratumoral administration of PFA/TPZ@UiO-66@PDA NPs or saline (scale bar, 50 μm). Asterisks indicate significant differences (*P < 0.05, **P < 0.01, ***P < 0.001, **** P < 0.0001)

To explore the treatment mechanism, 143B cells were grouped as follows: Control, TPZ, TPZ@UiO-66, and TPZ/PFA@UiO-66@PDA. After 3 h of incubation, RNA was extracted for RT-PCR analysis. Compared to the control group, HIF-1α, HIF-1β, p300, and CBP in the oxygen-dependent HIF-1α pathway were upregulated in the TPZ/PFA@UiO-66@PDA group, as were proapoptotic protein genes in the BCL-2 family (BAX, BAK, BID) and Caspase-3 (Fig. 4a). Western blotting showed that HIF-1α expression in the TPZ/PFA@UiO-66@PDA-treated group was higher than that in the control group (Fig. 4b). Immunohistochemistry of the hypoxyprobe revealed more positive cells in the TPZ/PFA@UiO-66@PDA-treated group, which also confirmed the enhanced cellular hypoxia (Fig. 4c). Next, we performed HIF-1α and Caspase-3 staining after 3 h of incubation. HIF-1α and Caspase-3 fluorescence was significantly stronger in the TPZ/PFA@UiO-66@PDA-treated group than in the other groups (Fig. 4d). Additional file 8: Fig. S7 showed 143B cells treated with 808nm laser demonstrated no upregulation of the oxygen-dependent HIF-1α pathway (HIF-1α, HIF-1β, p300, CBP) at the RNA level, and no upregulation of HIF-1α at the protein level. To study the in vivo efficacy of TPZ/PFA@UiO-66@PDA-induced hypoxia enhancement, 100 µL of TPZ/PFA@UiO-66@PDA NPs was intratumorally injected into 143B tumor-bearing mice. Photoacoustic images (Fig. 4e) showed that the average O_2_ content of tumor tissues continued to decrease after treatment with TPZ/PFA@UiO-66@PDA NPs for 0.5, 1, 2, and 3 h. In vivo photoacoustic imaging demonstrated that no decrease of O_2_ level was observed in the 808nm laser treated subcutaneous tumor mice models (Additional file 8: Fig. S7). Twenty-four hours after injection, the mice were sacrificed, and the tumors were sectioned for HIF-1α staining. HIF-1α fluorescence was significantly stronger in the TPZ/PFA@UiO-66@PDA-injected group than in the saline group (Fig. 4f).

### TPZ/PFA@UiO-66@PDA can suppress tumor growth effectively in vivo

**Fig. 5:**
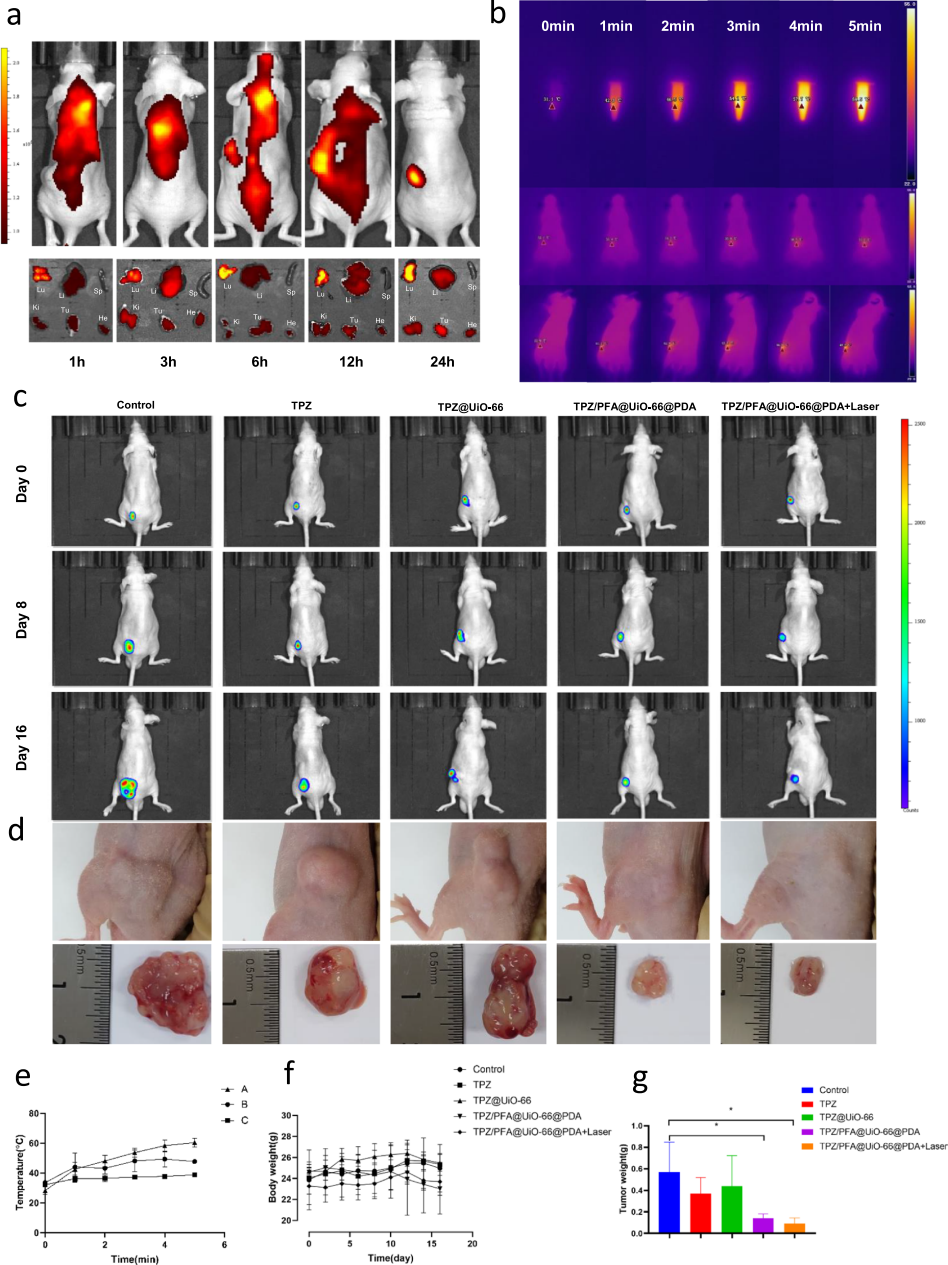
**a** In vivo fluorescence images of tumor-bearing mice and ex vivo tissue imaging after intravenous injection of IR780-doped PFA/TPZ@UiO-66@PDA NPs at different time points. He: heart, Li: liver, Sp: spleen, Lu: lung, Ki: kidney and Tu: tumor. **b** Temperature change and **e** the quantitative results at different time points in PFA/TPZ@UiO-66@PDA NPs and 143B tumor-bearing mice at 24 h after intravenous injection of saline or under laser irradiation (5 min, 808 nm, 1.5 W cm^− 2^) (n = 3). A, B, and C represent PFA/TPZ@UiO-66@PDA NPs, 143B tumor-bearing mice at 24 h after intravenous injection of PFA/TPZ@UiO-66@PDA NPs and 143B tumor-bearing mice at 24 h after intravenous injection of saline, respectively. **c** In vivo images of the subcutaneous tumor luciferase signal on day 0, day 8 and day 16. **d** Photographs of 143B tumor tissues in different groups after 16 days of treatment. **f** Body weight and **g** tumor weight of mice in different groups. Asterisks indicate significant differences (*P < 0.05, **P < 0.01, ***P < 0.001, **** P < 0.0001)

To evaluate the targeting ability and biodistribution of TPZ/PFA@UiO-66@PDA NPs after intravenous injection, 6-week-old male BALB/c 143B tumor-bearing mice were administered treatment via tail vein injection. After intravenous injection of IR-780-doped NPs into 143B tumor-bearing mice, accumulated fluorescence was observed, and major organs, including the tumor, heart, lung, liver, spleen, and kidney, were removed and observed at 1 h, 3 h, 6 h, 12 and 24 h. As shown in Fig. 5a, NPs accumulated at the tumor site 24 h after injection due to the EPR effect, and the fluorescence intensity in the tumors increased. Next, we studied the photothermal effect of TPZ/PFA@UIO-66@PDA NPs both in vitro and in vivo. The temperature of 500 µL of NPs (0.1 g/mL) increased to 60.27 ± 3.02 °C after 5 min under 808 nm laser irradiation. After 5 min under 808 nm laser irradiation, the temperature of the tumors in mice receiving a tail vein injection of 100 µL of NPs reached 47.67 ± 0.76 °C, which was higher than that in the control group receiving a tail vein injection of 100 µL of saline (38.67 ± 1.33 °C) (Fig. 5b and e). Figure 5c showed that the luminescence intensity in the subcutaneous tumors in the TPZ/PFA@UiO-66@PDA- and TPZ/PFA@UiO-66@PDA + laser-treated groups on day 8 and day 16 decreased compared with other groups. Figure 5f showed that the body weights of the mice in all groups remained within the normal range. The dissection of representative tumors was depicted in Fig. 5d. Smaller tumors were obtained from the TPZ/PFA@UiO-66@PDA and TPZ/PFA@UiO-66@PDA + laser groups, confirming the excellent antitumor ability of the TPZ/PFA@UiO-66@PDA NPs. The tumor weights in the TPZ/PFA@UiO-66@PDA- and TPZ/PFA@UiO-66@PDA + laser-treated groups were significantly smaller than those in the other groups (Fig. 5 g).

**Fig. 6:**
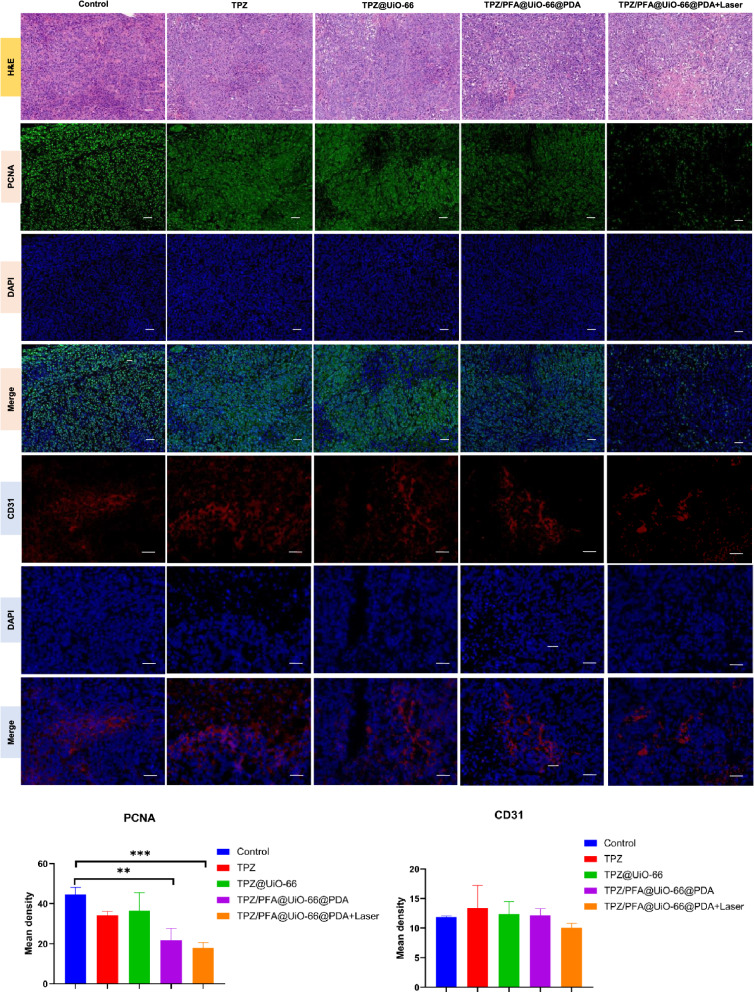
H&E staining of subcutaneous xenograft tumors and the expression of PCNA and CD31 in nude mouse tumor specimens in different groups analyzed by immunofluorescence (scale bar, 100 μm). Asterisks indicate significant differences (*P < 0.05, **P < 0.01, ***P < 0.001, **** P < 0.0001)

**Fig. 7:**
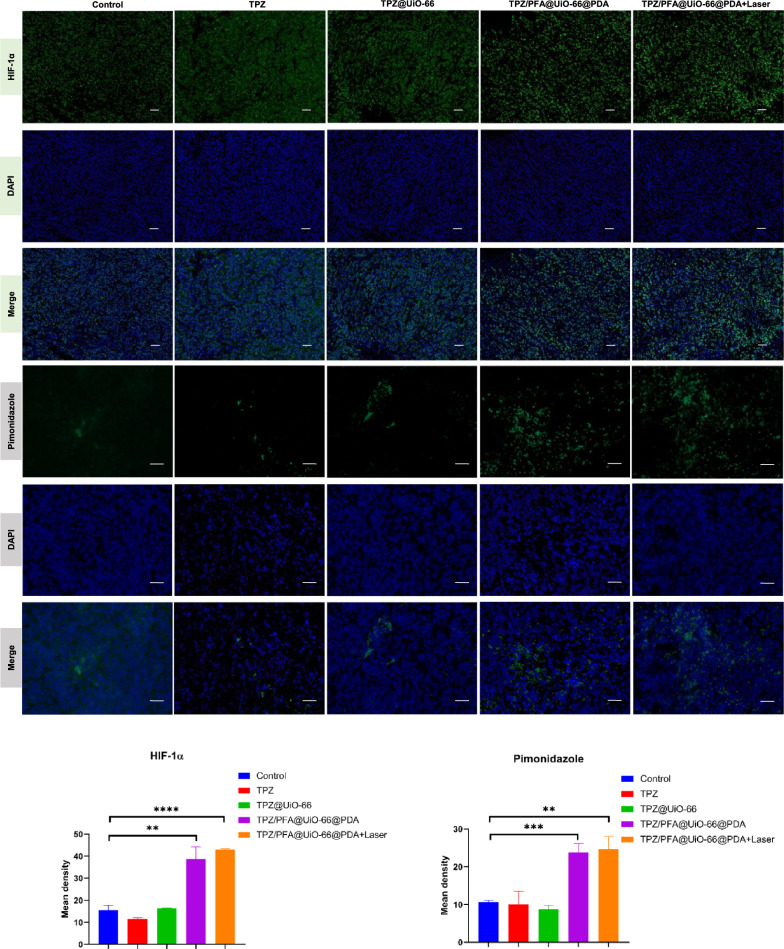
The expression of HIF-1α and pimonidazole in nude mouse tumor specimens in different groups analyzed by immunofluorescence (scale bar, 100 μm). Asterisks indicate significant differences (*P < 0.05, **P < 0.01, ***P < 0.001, **** P < 0.0001)

**Fig. 8:**
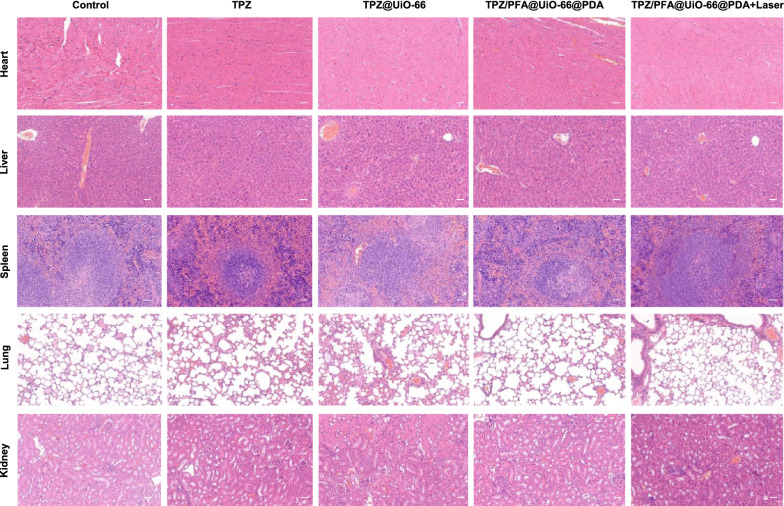
H&E staining of the major organs acquired from 143B tumor-bearing mice on day 16 with different treatments (scale bar, 100 μm)

**Fig. 9:**
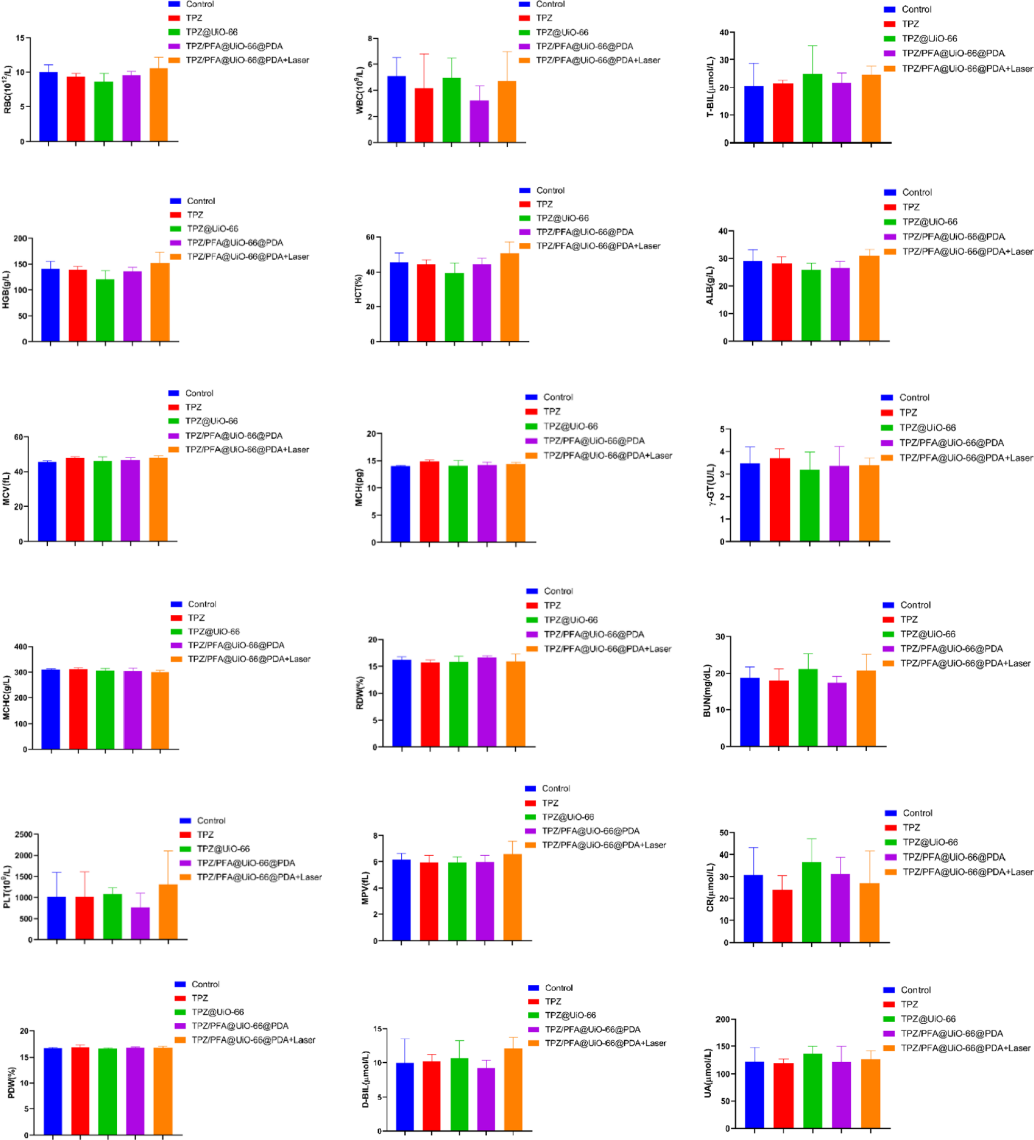
Blood biochemical levels and hematological parameters in 143B tumor-bearing mice on day 16 after different treatments

After treatment for 16 days, all mice were sacrificed, and the tumors were harvested and sectioned for H&E staining and immunohistochemistry of PCNA, CD31, HIF-1α and pimonidazole. Figure 6 demonstrated that PCNA fluorescence was significantly lower in the tumors treated with TPZ/PFA@UiO-66@PDA and TPZ/PFA@UiO-66@PDA + laser, whereas the fluorescence intensity of CD31 showed no difference among the groups. HIF-1α and pimonidazole fluorescence was significantly stronger in the TPZ/PFA@UiO-66@PDA- and TPZ/PFA@UiO-66@PDA + laser-treated groups, which indicated that TPZ/PFA@UiO-66@PDA NPs enhanced the hypoxic tumor environment but did not promote angiogenesis (Fig. 7). Moreover, these NPs showed excellent biocompatibility, since no obvious physiological morphological changes were observed in the major organs (liver, heart, lung, spleen and kidney) in any of the groups (Fig. 8), and various blood tests were within normal ranges (Fig. 9).

### Discussion

Hypoxia is a common feature in fast-growing solid tumors that hinders the effect of oxygen-dependent therapy, such as photodynamic therapy (PDT) and sonodynamic therapy (SDT), because PDT/SDT is highly dependent on the oxygen level and limited reactive species can be generated under hypoxia. Two major strategies have been developed to alleviate tumor hypoxia: increasing the O_2_ supply or reducing O_2_ consumption. However, most of these solutions have unsatisfactory therapeutic effects due to low intratumoral drug penetration. As the PA imaging in this study indicates, the rich blood supply around the tumor and insufficient vascularization inside the tumor tissue restrict these drugs to the normoxic tumor periphery rather than the hypoxic core.

Cancer starvation therapy is the concept of depriving tumors of nutrient supply to suppress tumor growth. In parallel with conventional vascular occlusion methods, intratumoral glucose/oxygen depletion nanotechnology has emerged and developed rapidly. For example, magnesium silicide NPs can remove intratumoral oxygen efficiently to starve cancer cells [35]. Shan et al. [36] constructed an antioxidant nanocarrier coencapsulating glucose oxidase (GO) and TPZ, which can create tumor hypoxia to convert TPZ into toxic radicals via rapid oxygen depletion while protecting normal tissues by scavenging GOx-catalyzed aerobic glycometabolism byproducts. Perfluorocarbon (PFC) is a promising artificial blood substitute. PFC is also a tumor-specific oxygen carrier due to its excellent oxygen solubility and has been used to relieve tumor hypoxia and enhance the therapeutic effect of PDT/SDT [37]. Liang et al. [38] demonstrated that PFC@porphyrin NPs could serve as an important oxygen reservoir and relieve tumor hypoxia to enhance PDT. Sun et al. [39] developed a mesenchymal stem cell membrane-functionalized liposomal formulation of the sonosensitizer verteporfin and oxygen-loaded PFC, resulting in enhanced SDT therapeutic effects. Although PFC has a high affinity for oxygen, its oxygen release efficacy is quite low. In this study, PFC was used as an intratumoral oxygen-consuming material rather than an oxygen delivery material. In addition, Wang et al. [16] reported that PFC NPs can be used to create a long-lasting, hypoxic intratumoral environment to enhance the effect of hypoxia-based prodrugs. MOF NPs are characterized by their high porosity, large surface area and high thermal stability. UiO-66 is a porous zirconium-based MOF with open octahedral and tetrahedral cavities [40], and it has been developed for many applications, such as drug delivery systems [41–43], biosensors [44, 45], gas/heavy metal absorbents [33, 46], and biomedicine. In this study, we used UiO-66 as a drug-loading platform by coencapsulating PFA and TPZ, and a PDA coating was added to prevent premature drug leakage.

Notably, it is widely accepted that tumor hypoxia can promote tumor neovascularization and metastasis. In this study, CD31 was not upregulated in groups 4 and 5. However, whether aggravated tumor hypoxia promotes angiogenesis in the long term remains unknown. Moreover, 143B is not a highly metastatic cell line, and it is necessary to repeat this study with a highly metastatic osteosarcoma cell line to confirm that a tumor deoxygenation treatment strategy would not accelerate malignant tumor metastasis. Second, large numbers of NPs face immune clearance when injected intravenously. Various surface functionalization methods have been explored to enhance the utility of NPs. For example, Zhang et al. [27] reported an erythrocyte membrane-cloaked MOF-based nanomaterial to reduce NP elimination by the immune system. Sun et al. [39] constructed a mesenchymal stem cell membrane-functionalized biomimetic sonosensitizer, which increased circulation and targeting efficacy. Further studies should focus on enhancing the permeability and retention effect of nanomaterials in the tumor while reducing immune clearance. Another challenge is the limited penetration of 808 nm laser light, as osteosarcoma is a nonsuperficial disease. Some progress has been made toward solving this problem, including strategies such as converting near-infrared rays into photodynamic light in situ [47–50] and taking advantage of aggregation-induced emission photosensitizers to achieve strong radiotherapy and radiodynamic therapy effects under X-ray irradiation [51]. In addition, strong fluorescence intensity was observed in the lungs 24 h after tail vein injection, which could be a concern in this study.

## Conclusions

In summary, we successfully constructed biocompatible TPZ/PFA@UiO-66@PDA NPs to suppress tumor growth by enhancing the hypoxic tumor environment. Once injected into the tail vein, TPZ/PFA@UiO-66@PDA NPs accumulated in the tumor region via the EPR effect. After uptake by tumor cells, TPZ/PFA@UiO-66@PDA NPs were degraded to release PFA, TPZ and PDA. Then, PFA upregulated the oxygen-dependent HIF-1α pathway and induced apoptosis by enhancing the hypoxic tumor environment. TPZ transformed to toxic BTZ and was activated by enhanced tumor hypoxia. PTT was utilized to further enhance drug-resistant tumor cell killing. Consequently, tumor growth was significantly suppressed (Additional files 1, 2, 3, 4, 5, 6, 7, 8).

## Supplementary Information

**Additional file 1. **Supplementary Methods.

**Additional file 2: Fig. S1. **PXRD spectra of UiO-66.

**Additional file 3: Fig. S2. **SEM and TEM images of TPZ/PFA@UiO-66@PDA.

**Additional file 4: Fig. S3. **SEM images of TPZ/PFA@UiO-66@PDA dispersed in culture media or PBS for 24 h.

**Additional file 5: Fig. S4. **The standard calibration curve of TPZ and the in vitro release curve of TPZ from TPZ@UiO-66 and TPZ/PFA@UiO-66@PDA.

**Additional file 6: Fig. S5. **Cytotoxicity of UiO-66 in 143B and R-BMSC after co-incubation for 3 days.

**Additional file 7: Fig. S6. **Cell viability of 143B cells after incubation with different treatments for 3 days.

**Additional file 8: Fig. S7. **Influence of the 808 nm laser on the oxygen-dependent HIF-1α pathway in vitro at the RNA and protein level and the average oxygen content of tumor tissues in vivo

## Data Availability

All data generated or analyzed during this study are included in this manuscript.

## Abbreviations

PFA: Perfluorotributylamine
TPZ: Tirapazamine
PDA: Polydopamine
MOF: Metal organic framework
PTT: Photothermal therapy
BTZ: Benzotriazinyl
XRD: X-ray diffraction
SEM: Scanning electron microscopy
TEM: Transmission electron microscopy
TGA: Thermogravimetric analysis
DMEM: Dulbecco’s minimum essential medium
GAPDH: Glyceraldehyde 3-phosphate dehydrogenase 1
PA: Photoacoustic
H&E: Hematoxylin-eosin
SD: Standard deviation
ANOVA: One-way analysis of variance
PDT: Photodynamic therapy
SDT: Sonodynamic therapy
GO: Glucose oxidase
PFC: Perfluorocarbon
EPR effect: Enhanced permeability and retention effect
