# Supplementary material for: Polydopamine-coated UiO-66 nanoparticles loaded with perfluorotributylamine/tirapazamine for hypoxia-activated osteosarcoma therapy
Source: J Nanobiotechnology. 2021 Sep 30;19:298. doi: 10.1186/s12951-021-01013-0 (PMC8482624; doi:10.1186/s12951-021-01013-0)
Supplement: Supplementary file 1 — Additional file 1. Supplementary Methods. [file 12951_2021_1013_MOESM1_ESM.docx]

**Supplementary Fig. 1** PXRD spectra of UiO-66.

**Supplementary Fig. 2** SEM and TEM images of TPZ/PFA@UiO-66@PDA.

**Supplementary Fig. 3** SEM images of TPZ/PFA@UiO-66@PDA dispersed in culture media or PBS for 24 h.

**Supplementary Fig. 4** The standard calibration curve of TPZ and the in vitro release curve of TPZ from TPZ@UiO-66 and TPZ/PFA@UiO-66@PDA.

**Supplementary Fig. 5** Cytotoxicity of UiO-66 in 143B and R-BMSC after co-incubation for 3 days.

**Supplementary Fig. 6** Cell viability of 143B cells after incubation with different treatments for 3 days.

**Supplementary Fig. 7** Influence of the 808nm laser on the oxygen-dependent HIF-1α pathway *in vitro* at the RNA and protein level and the average oxygen content of tumor tissues *in vivo*.

**Supplementary Methods**

General characterization

The crystallinity of the prepared UiO-66 MOF were detected using Powder X-ray diffraction (PXRD, D8 VENTURE). Surface morphology of the TPZ/PFA@UiO-66@PDA nanoparticles were observed using scanning electron microscopy (SEM, MIRA3), and transmission electron microscopy (TEM, Talos F200X G2). The in vitro release of TPZ from TPZ@UiO-66 and TPZ/PFA@UiO-66@PDA were calculated by using the standard calibration curve based on the UV-vis absorption intensity at 470 nm^1^.

Cell culture

143B cells were obtained from ATCC (Manassas, VA) and used in this study. Rat bone marrow stem cells (R-BMSC) were extracted from Sprague Dawley rats. Cells were cultured in Dulbecco's minimum essential medium (DMEM) supplemented with 10% fetal bovine serum (FBS), penicillin (100 U/mL), and streptomycin (100 mg/mL) in a humidified atmosphere containing 5% CO_2_ at 37°C. The culture medium was changed every 2 days. Cells were passaged upon reaching 80-90% confluence.

Cell viability

Cell viability was assessed using Cell Counting Kit-8 method. For UiO-66 toxicity test, 143B cells and R-BMSC were cultured with UiO-66 (0 µg mL^-1^, 10 µg mL^−1^, 20 µg mL^-1^, 40 µg mL^-1^, 80 µg mL^-1^). For 143B cells, cells were cultured in 96-well plates at a density of 1×10^3^ cells/well for 3 days. For R-BMSC, cells were cultured in 96-well plates at a density of 5×10^3^ cells/well for 3 days. Then 143B cells were divided into the following groups: control, TPZ (40 µg mL^-1^), TPZ@UiO-66 (40 µg mL^-1^), 808 nm laser, TPZ/PFA@UiO-66@PDA (40 µg mL^-1^), and TPZ/PFA@UiO-66@PDA+808 nm laser radiation. Each group was cultured for 3 days in 96-well plates at a density of 5×10^3^ cells/well. For group 4 and 6, cells were irradiated with an 808 nm laser for 5 min.

Quantitative real-time PCR

To examine the effects of the 808nm laser on oxygen-dependent signaling in 143B cells at the gene level, 143B cells were seeded and grouped as Control and Laser (1.5 W cm^-2^, 5 min). Both groups were cultured in 6-well plates at a density of 1×10^5^ cells/well for 3 h. Total RNA was isolated using an E.Z.N.A.® HP Total RNA Kit (Omega Bio-Tek, Norcross, GA, USA). One microgram of RNA was reverse transcribed using a Prime-Script RT reagent kit (Takara, Shiga, Japan). Real-time PCR was performed in a 7300 Real-Time PCR system using SYBR® Premix Ex Taq^TM^ (Takara, Shiga, Japan). Gene expressions of HIF-1α, HIF-1β, p300 and CBP were detected in this section. Glyceraldehyde 3-phosphate dehydrogenase 1 (GAPDH) was evaluated for each RT reaction as a standard. The ΔΔCT method was used to analyze relative gene expression.

Western blotting analysis

To examine the effects of 808nm laser on HIF-1α in 143B cells at the protein level, 143B cells were seeded and grouped as Control and Laser (1.5 W cm^-2^, 5 min). Both groups were cultured for 3 h in 6-well plates at a density of 1×10^5^ cells/well. The cells were washed three times with PBS and then lysed with RIPA buffer (150 mM NaCl, 1% sodium deoxycholate, 0.1% SDS, 50 mM Tris-HCl pH 7.4, 1 mM EDTA, 1 mM PMSF, and 1% Triton X-100) supplemented with protease inhibitors and phosphatase inhibitors for 30 min at 4 °C. A total of 20-30 mg of protein was separated via 10% sodium dodecyl sulfate-polyacrylamide gel electrophoresis and electrotransferred onto nitrocellulose membranes. A rabbit polyclonal anti-HIF-1α antibody (Cell Signaling Technology) was used as the primary antibody. β-Actin antibody (Cell Signaling Technology) was used to normalize protein loading. The protein bands were visualized using an Odyssey Infrared Imaging System (LI-COR Biosciences, Lincoln, NE, USA).

*In vivo* photoacoustic (PA) imaging

For *in vivo* PA imaging, 143B tumor-bearing mice were irradiated by laser (808 nm, 1.5 W cm^-2^) for 5 min. Then intensities of PA signals and PA images of tumor-bearing mice were measured and recorded at different time points (0, 0.5 and 1 h). The imaging parameters were set as follows: PA gain, 47 dB, and 2D gain, 20 dB.

**References:**

1. Zhang L, Wang Z, Zhang Y, Cao F, Dong K, Ren J, Qu X. Erythrocyte Membrane Cloaked Metal-Organic Framework Nanoparticle as Biomimetic Nanoreactor for Starvation-Activated Colon Cancer Therapy. ACS Nano. 2018 Oct 23;12(10):10201-10211.
