# Supplementary figures and images for: Polydopamine-coated UiO-66 nanoparticles loaded with perfluorotributylamine/tirapazamine for hypoxia-activated osteosarcoma therapy
Source: J Nanobiotechnology. 2021 Sep 30;19:298. doi: 10.1186/s12951-021-01013-0 (PMC8482624; doi:10.1186/s12951-021-01013-0)

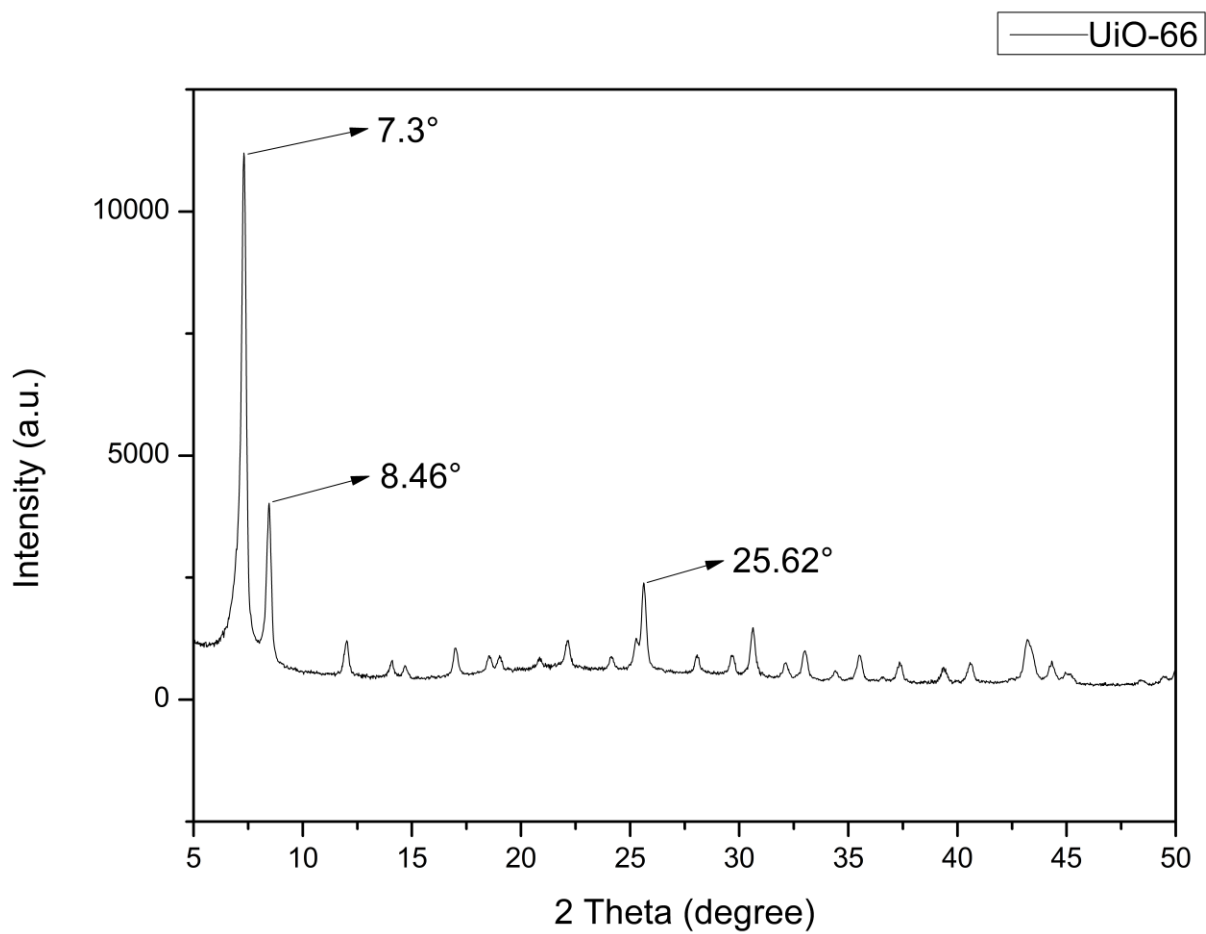

Supplement: Supplementary file 2 — Additional file 2: Fig. S1. PXRD spectra of UiO-66. [file 12951_2021_1013_MOESM2_ESM.pdf]

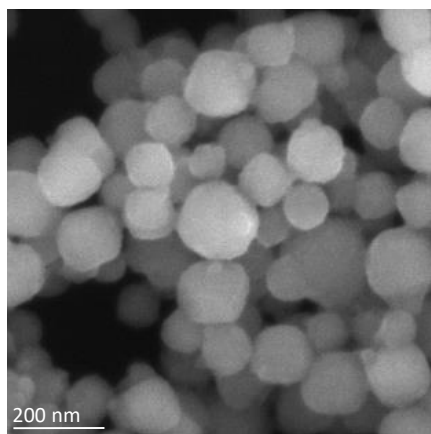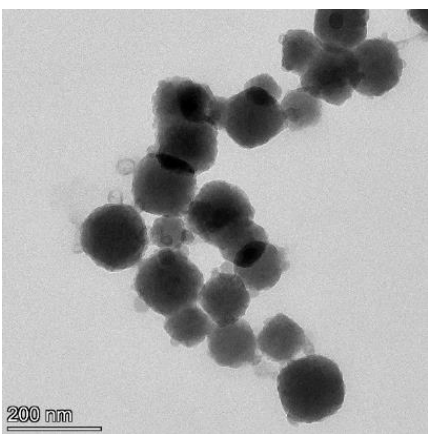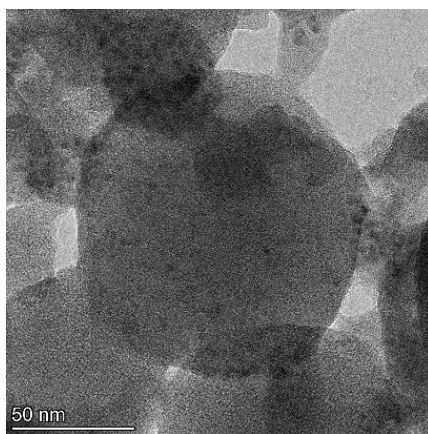

Supplement: Supplementary file 3 — Additional file 3: Fig. S2. SEM and TEM images of TPZ/PFA@UiO-66@PDA. [file 12951_2021_1013_MOESM3_ESM.pdf]

**Culture media**

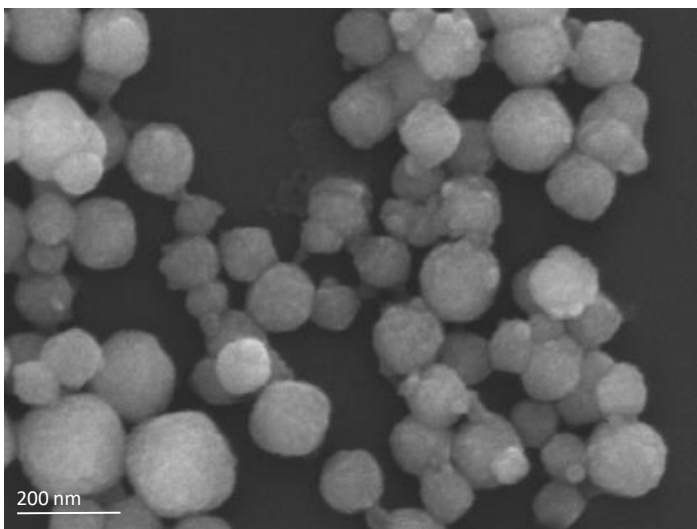

**PBS**

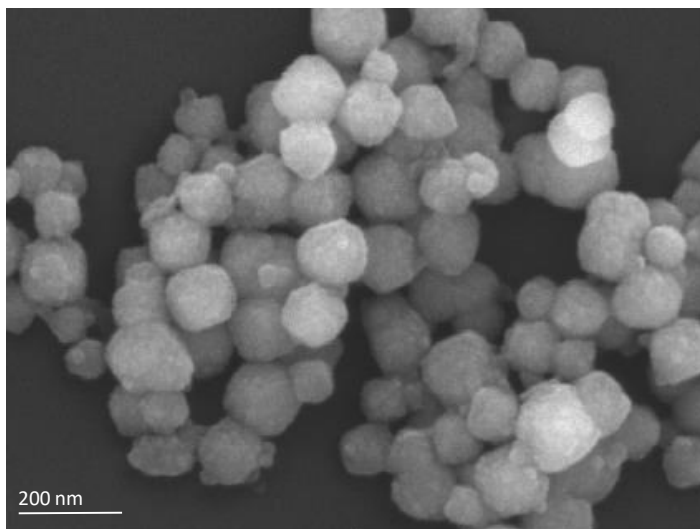

Supplement: Supplementary file 4 — Additional file 4: Fig. S3. SEM images of TPZ/PFA@UiO-66@PDA dispersed in culture media or PBS for 24 h. [file 12951_2021_1013_MOESM4_ESM.pdf]

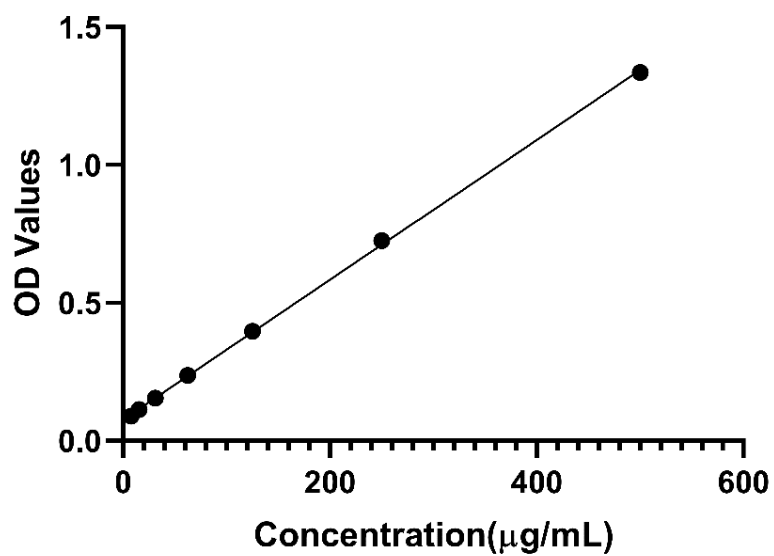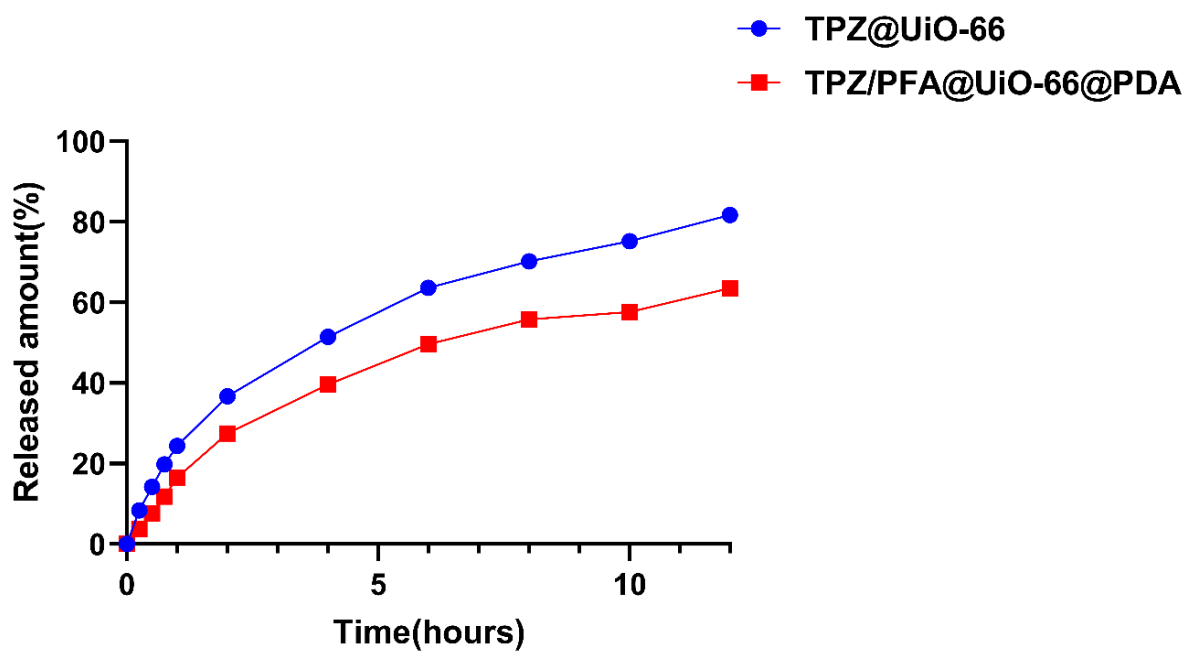

Supplement: Supplementary file 5 — Additional file 5: Fig. S4. The standard calibration curve of TPZ and the in vitro release curve of TPZ from TPZ@UiO-66 and TPZ/PFA@UiO-66@PDA. [file 12951_2021_1013_MOESM5_ESM.pdf]

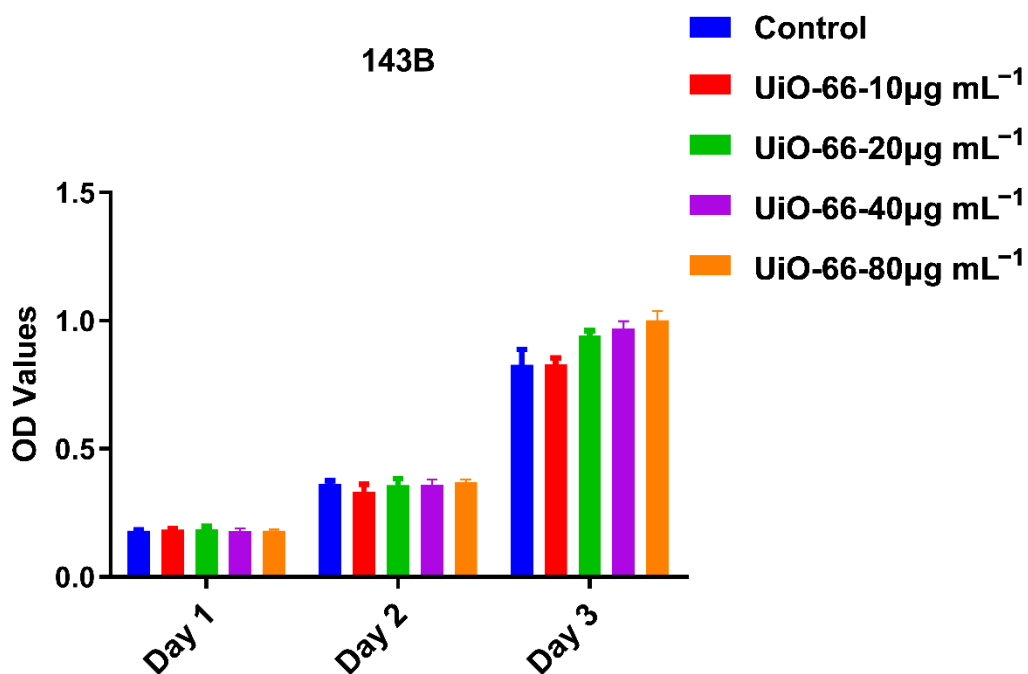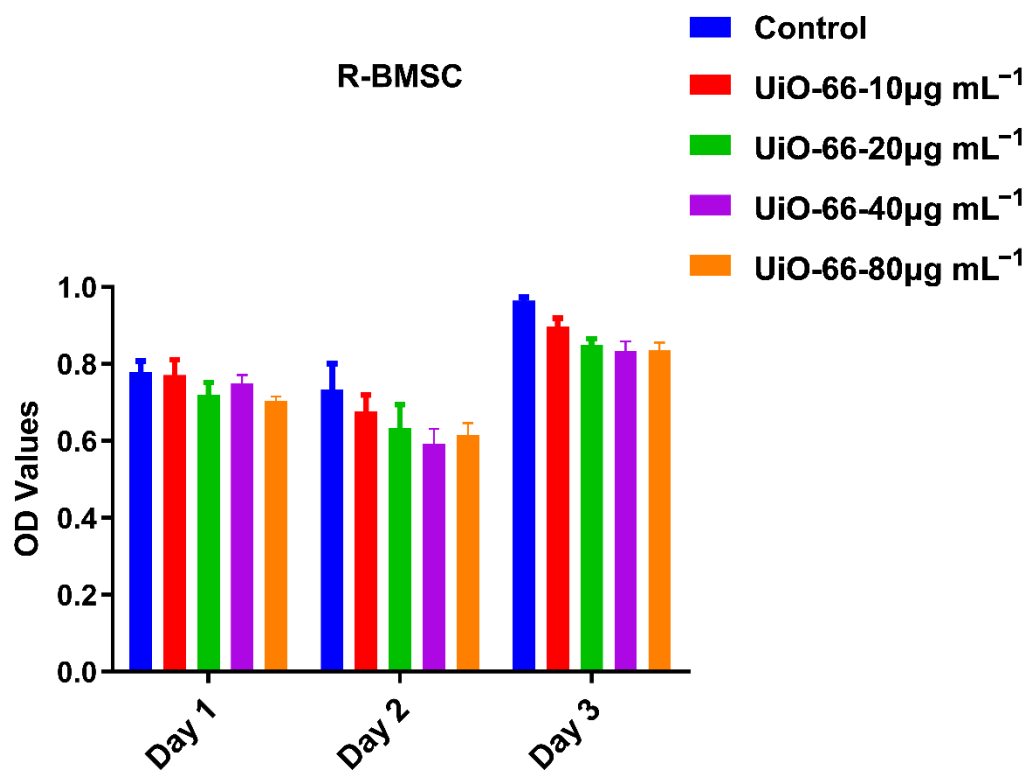

Supplement: Supplementary file 6 — Additional file 6: Fig. S5. Cytotoxicity of UiO-66 in 143B and R-BMSC after co-incubation for 3 days. [file 12951_2021_1013_MOESM6_ESM.pdf]

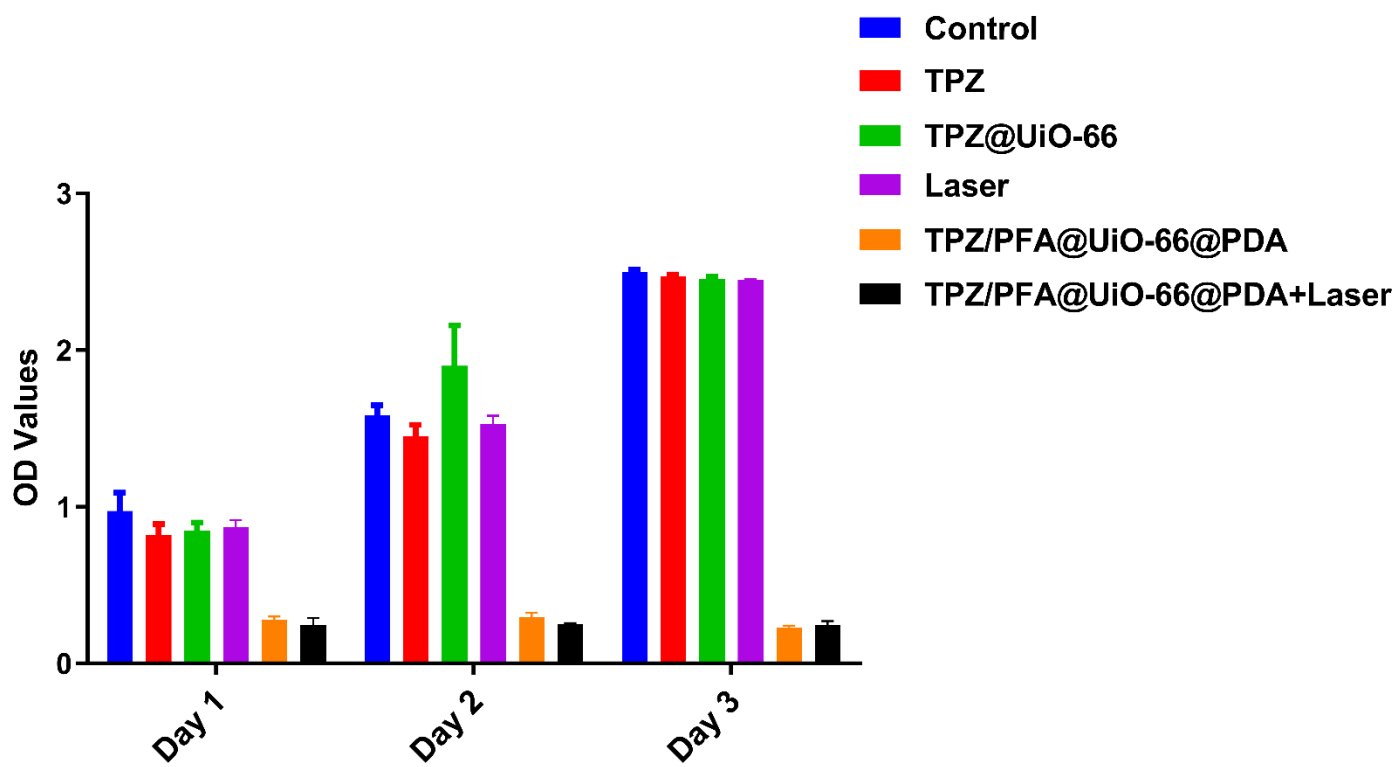

Supplement: Supplementary file 7 — Additional file 7: Fig. S6. Cell viability of 143B cells after incubation with different treatments for 3 days. [file 12951_2021_1013_MOESM7_ESM.pdf]

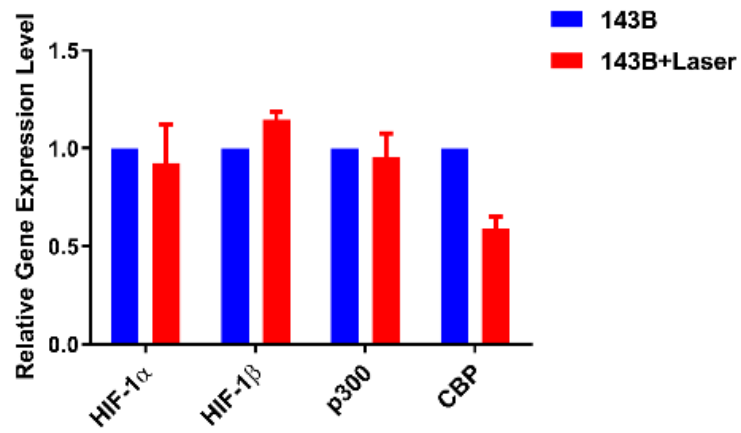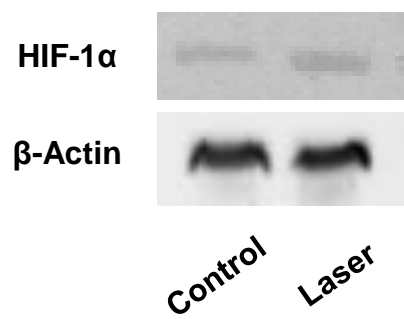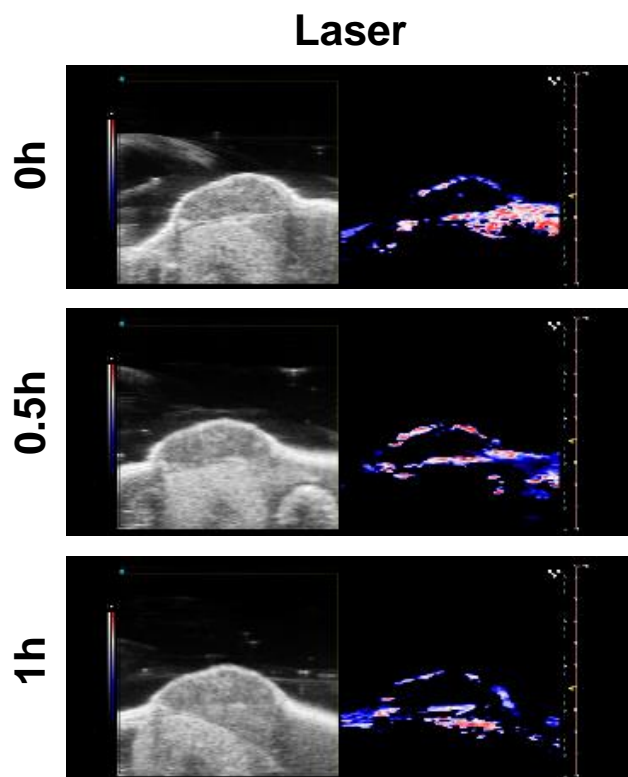

Supplement: Supplementary file 8 — Additional file 8: Fig. S7. Influence of the 808 nm laser on the oxygen-dependent HIF-1α pathway in vitro at the RNA and protein level and the average oxygen content of tumor tissues in vivo [file 12951_2021_1013_MOESM8_ESM.pdf]
